# Supplementary material for: Aqueous Extract of Phyllanthus emblica L. Alleviates Functional Dyspepsia through Regulating Gastrointestinal Hormones and Gut Microbiome In Vivo
Source: Foods. 2022 May 20;11(10):1491. doi: 10.3390/foods11101491 (PMC9141879; doi:10.3390/foods11101491)
Supplement: Supplementary file 1 [file foods-11-01491-s001.zip › foods-1651339-supplementary.pdf]

Supplementary Material

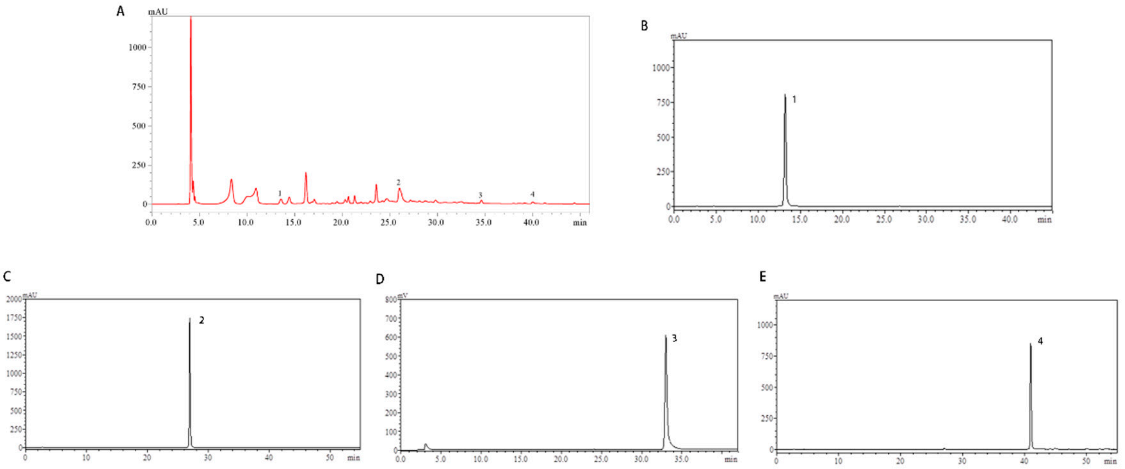

Figure **S1** Representative high-performance liquid chromatograms of APE sample (**A**) and standard compounds (**B–E**). 1, Gallic acid; 2, corilagin; 3, ellagic acid; and 4, fistin.
